# Decreasing Frequency Splits of Hemispherical Resonators by Chemical Etching

**DOI:** 10.3390/s18113772

**Published:** 2018-11-05

**Authors:** Yuting Wang, Yao Pan, Tianliang Qu, Yonglei Jia, Kaiyong Yang, Hui Luo

**Affiliations:** College of Advanced Interdisciplinary Studies, National University of Defense Technology, Deya 109, Changsha 410073, China; wangwangnamely@foxmail.com (Y.W.); yaomeredithpan@hotmail.com (Y.P.); jiayl2016@163.com (Y.J.); yky208@nudt.edu.cn (K.Y.); luohui.luo@163.com (H.L.)

**Keywords:** hemispherical resonator, frequency split, chemical etching

## Abstract

The hemispherical resonator gyroscope (HRG) has attracted the interest of the world inertial navigation community because of its exceptional performance, ultra-high reliability and its potential to be miniaturized. These devices achieve their best performance when the differences in the frequencies of the two degenerate working modes are eliminated. Mechanical treatment, laser ablation, ion-beams etching, etc., have all been applied for the frequency tuning of resonators, however, they either require costly equipment and procedures, or alter the quality factors of the resonators significantly. In this paper, we experimentally investigated for the first time the use of a chemical etching procedure to decrease the frequency splits of hemispherical resonators. We provide a theoretical analysis of the chemical etching procedure, as well as the relation between frequency splits and mass errors. Then we demonstrate that the frequency split could be decreased to below 0.05 Hz by the proposed chemical etching procedure. Results also showed that the chemical etching method caused no damage to the quality factors. Compared with other tuning methods, the chemical etching method is convenient to implement, requiring less time and labor input. It can be regarded as an effective trimming method for obtaining medium accuracy hemispherical resonator gyroscopes.

## 1. Introduction

The hemispherical resonator gyroscope (HRG) is a kind of Coriolis vibratory gyroscope (CVG) well-known for its high performance, small size, light weight, low power consumption and maintenance-free concept [1]. These qualities make HRGs well adapted for space and marine applications such as spacecraft stabilization, precision pointing, attitude and heading reference systems, inertial navigation systems and planetary exploration [1,2]. The hemispherical resonator is the key element of the HRG to detect the angle of rotation, which determines the overall performance of the HRG. Hemispherical resonators work at *n* = 2 resonant mode. Two principle axes have their own oscillation frequencies *f*_1_ and *f*_2_, and they are equal ideally. However, the shell thickness and surface state of the machine-made resonators are not perfect, which results in stiffness and mass distribution variations and causes differences between *f*_1_ and *f*_2_. Frequency split is defined as ∆*f* = |*f*_1_ − *f*_2_|, which is one of the main error sources in vibratory gyroscopes [3]. Some researchers have applied electrostatic forces to control the frequency splits [4,5,6,7], however, the inaccuracies of the electronics and actuators would introduce extra errors. Therefore, it is preferred to eliminate the frequency split during the manufacture of the resonator [8].

Many theoretical studies have focused on the frequency split of the resonator. Fox [9] proposed that to eliminate the frequency split, a trimming mass must be either added to the antinodes of the higher-frequency axis or removed from the antinodes of the lower-frequency axis. Rourke et al. [10,11] extended and generalized the trimming procedure and the results of Fox [9], using more than one trimming mass to adjust more than one pair of modes. They also studied the effect of mass imperfections on the natural frequencies and orientations of a perfect ring, and proposed a method to eliminate frequency splits between a pair of in-plane modes and that between a pair of out-of-plane modes of an imperfect ring. Schwartz et al. [12] developed a mass matrix perturbation approach for a disk resonator gyro to tune two degenerate modes, where small magnets were employed to induce perturbations to the resonator mass. Choi et al. [13] put forward an approach to determine the natural frequency of a hemispherical shell, and investigated the frequency split variation for different thicknesses and radii. Zhen et al. [14] studied the frequency split of an axisymmetric multi-curved surface shell resonator, and their approach could also be used on other kinds of resonators with limited precision. Jung-Hwan et al. [15] derived the analytical model of the eigenfrequency split on an imperfect hemispherical shell, and established a function to predict the trimming mass. Matveev et al. [16] deduced a formula of the relationship between the unbalanced mass and the frequency split, which could be used to analytically predict the trimming mass. Those theoretical analyses provide a basic understanding of the mechanism for reducing frequency splits of hemispherical resonators.

In practice, methods such as mechanical treatment, laser ablation, chemical etching, ion-beams etching, etc., have all been applied for the frequency tuning of resonators. Yi et al. [17] reported their work on mechanical balancing for a cupped wave gyro based on cup-bottom trimming, minimizing the natural frequency split to 10^−3^ grade. Hamelin et al. [18] demonstrated a mechanical eutectic trimming of a polysilicon microhemispherical resonating gyroscope using Joule heating and reduced the frequency split to 0.1 Hz. However, mechanical treatment might induce the unbalance of other physical properties, and might also degrade the quality factors of resonators [19]. Laser ablation is widely used in micromechanical vibrating resonators [20,21,22], and the target material is usually silicon. Kim et al. [23] used laser ablation of a conformal layer to expose silicon for mass removal by subsequent deep reactive ion retching (DRIE), reducing frequency splits to less than 0.1 Hz. As for HRG resonators, the most commonly used material is fused silica, which can greatly promote the quality factor [24,25]. However, fused silica is brittle in nature and thus not well suited for laser ablation or mechanical trimming [26]. Lynch [27] designed thirty-one extended tines beyond the equator of the hemispherical shell, and mass was removed from selected tines to eliminate the frequency mismatch. However, these tines greatly increased the difficulty and cost of manufacturing, and the unused tines might bring other unwanted results, therefore this design has been abandoned despite excellent results. Hu et al. [28] used ion beam to balance the mass of hemispherical resonators, and the frequency split was reduced from 0.46 Hz to 0.012 Hz. However, he also pointed out that this method consumed a large amount of time and energy, and was only able to adjust the frequency split within a small range. The conception and procedure of tuning resonators by chemical etching was first proposed by Basarab et al. [29]. However, until now there have been no reports of experimental results on reducing frequency splits of hemispherical resonators by chemical etching, because some technical difficulties still exist in conducting chemical etching experiments, such as temperature stability, small changes of chemical etching solution concentration, position accuracy of the four antinodes, the precise control of the etching height and inclined angle, etc. 

In this paper, decreasing frequency splits of hemispherical resonators by chemical etching is realized. Section 2 gives theoretical analyses on the relationship between mass errors and frequency splits of hemispherical resonators, and briefly presents the theoretical aspects of chemical balancing method according to Basarab et al. [29]. Section 3 shows the simulation results on an imperfect hemispherical resonator model obtained by the COMSOL Multiphysics. Section 4 demonstrates the procedure and results of chemical etching to decrease frequency splits of three hemispherical resonators, where quality factors are also considered. Frequency splits of three resonators were decreased from 5.529 Hz, 1.44 Hz and 0.805 Hz to 0.364 Hz, 0.025 Hz, and 0.049 Hz, respectively. Results were then compared with theoretical calculations considering the fourth harmonic error of the resonator mass. Chemical etching method showed its possibilities as an effective and cost-friendly tuning method, without damaging the quality factors.

## 2. Theoretical Analysis

**Figure 1:**
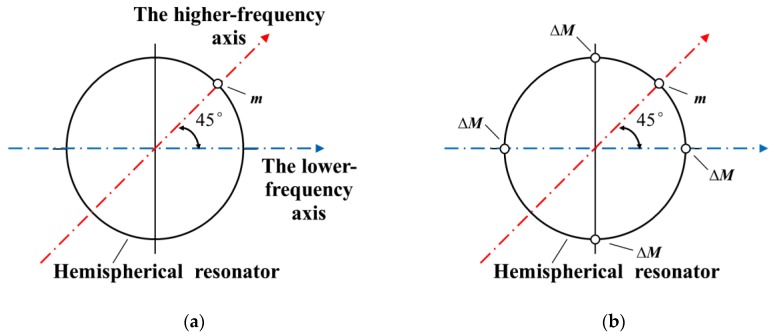
(**a**) One mass defect *m* appears on a hemispherical resonator, and is located along the higher-frequency axis. (**b**) Four mass points ∆*M* are removed symmetrically from the edge of a hemispherical resonator.

Consider a hemispherical resonator with a mass of *M*_0_ and having a natural frequency of *ω*_0_ under *n* = 2 mode. When one mass defect *m* appears on the edge, as shown in Figure 1a, according to Fox [9], it is located along the higher-frequency axis. The natural frequencies can be written as:

(1) $$\omega_{1}^{2}=\omega_{0}^{2}\left[\frac{\left(1+\alpha_{2}^{2}\right)M_{0}}{\left(1+\alpha_{2}^{2}\right)\left(M_{0}-m\right)-m(1-\alpha_{2}^{2})}\right],\;$$

(2) $$\omega_{2}^{2}=\omega_{0}^{2}\left[\frac{\left(1+\alpha_{2}^{2}\right)M_{0}}{\left(1+\alpha_{2}^{2}\right)\left(M_{0}-m\right)+m(1-\alpha_{2}^{2})}\right],\;$$

where *ω*_1_ and *ω*_2_ refers to the higher frequency and the lower frequency (*ω*_1_ ≥ *ω*_2_), and *α*_2_ refers to the ratio of the maximum radial displacement to the maximum tangential displacement. Combining Equations (1) and (2), the following equation is obtained:

(3) $$\frac{\omega_{0}^{2}}{\omega_{2}^{2}}-\frac{\omega_{0}^{2}}{\omega_{1}^{2}}=\frac{\omega_{0}^{2}(\omega_{1}+\omega_{2})(\omega_{1}-\omega_{2})}{\omega_{1}^{2}\omega_{2}^{2}}=\frac{2m(1-\alpha_{2}^{2})}{(1+\alpha_{2}^{2})M_{0}},\;$$

where *ω*_1_ − *ω*_2_ represents the frequency mismatch ∆*ω*. If the extra mass *m* is small, we can assume that:

(4) $$\omega_{1}+\omega_{2}\approx 2\omega_{0},\omega_{1}^{2}\approx \omega_{2}^{2}\approx \omega_{0}^{2}.\;$$

Thus, Equation (3) can be rewritten as:

(5) $$\frac{\Delta \omega }{\omega_{0}}=\frac{m(1-\alpha_{2}^{2})}{(1+\alpha_{2}^{2})M_{0}}.\;$$

In order to eliminate the frequency mismatch, four mass points (4∆*M*) are removed from the lower-frequency axis, according to Fox [9]. Similarly, the natural frequencies can be written as

(6) $${\omega^{\prime }}_{1}^{2}=\omega_{0}^{2}\left[\frac{\left(1+\alpha_{2}^{2}\right)M_{0}}{\left(1+\alpha_{2}^{2}\right)\left(M_{0}-m-4\Delta M\right)-(4\Delta M-m)(1-\alpha_{2}^{2})}\right],\;$$

(7) $${\omega^{\prime }}_{2}^{2}=\omega_{0}^{2}\left[\frac{\left(1+\alpha_{2}^{2}\right)M_{0}}{\left(1+\alpha_{2}^{2}\right)\left(M_{0}-m-4\Delta M\right)+(4\Delta M-m)(1-\alpha_{2}^{2})}\right],\;$$

Thus, the following equation is obtained:

(8) $$\frac{\Delta \omega^{\prime }}{\omega_{0}}=\frac{\left(m+4\Delta M)(1-\alpha_{2}^{2}\right)}{(1+\alpha_{2}^{2})M_{0}}=\frac{\Delta \omega }{\omega_{0}}-\frac{4\Delta M(1-\alpha_{2}^{2})}{(1+\alpha_{2}^{2})M_{0}}.\;$$

In order to make ∆*ω*’ equal to zero, the total mass to be removed (4∆*M*) must satisfy:

(9) $$4\Delta M=\frac{\Delta \omega }{\omega_{0}}\cdot \frac{(1+\alpha_{2}^{2})M_{0}}{\left(1-\alpha_{2}^{2}\right)}\;$$

For a specific hemispherical resonator, the amount of the mass to be removed or added can be calculated according to its initial frequency split, and the principle vibrating axes are easy to determine experimentally. Frequency split ∆*f* has a relationship with frequency mismatch ∆*ω* in the form:

(10) $$\Delta f=\frac{\Delta \omega }{2\pi }.\;$$

In combination with Equation (10), Equation (9) can be rewritten as:

(11) $$\frac{\Delta f}{4\Delta M}=\frac{(1-\alpha_{2}^{2})f_{0}}{(1+\alpha_{2}^{2})M_{0}}=K,\;$$

where *f*_0_ refers to the natural frequency. Equation (11) reveals that unbalanced mass is linearly related with the frequency split, *K* is here to indicate the slope. The simulation research of Jung-Hwa [15] suggested that the mass location was a significant factor that affected the slope *K*.

Basarab et al. [29] put up the chemical etching method. In his research, the resonator’s mass distribution *M*(*φ*) can be expressed in Fourier series with respect to the circumferential angle *φ*:

(12) $$M(\varphi )=M_{0}+\sum_{k=1}^{\infty }M_{k}\cos k(\varphi -\varphi_{k}),\;$$

where *M*_0_ is the uniformly distributed mass in the circumferential angle; *k* is the harmonic number of the Fourier series; *M_k_* is the *k*-th harmonic of the resonator mass, while *φ_k_* is the corresponding orientation of *M_k_* with respect to the defined zero direction.

**Figure 2:**
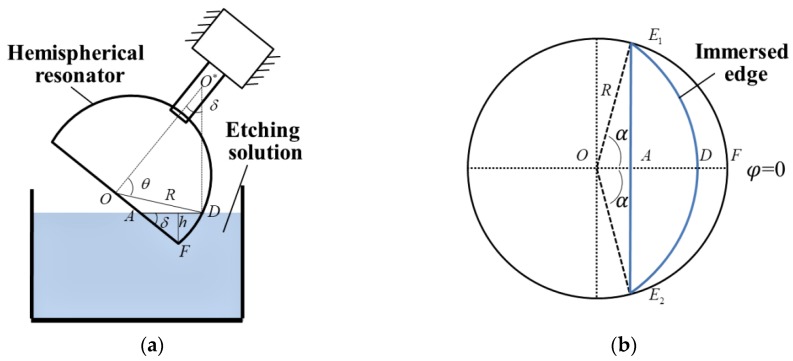
Schematic of main parameters in chemical etching procedure. (**a**) The hemispherical resonator with radius *R* is immersed into the etching solution, with an immersion depth *h* and an inclination angle *δ*; (**b**) The immersed edge is projected onto the plane of the resonator cup [29], the zenith angle of which is *α*.

This paper mainly focuses on reducing *M*_4_, which is the cause of the frequency split of the resonator and the main sources of bias and random errors in HRGs. According to Basarab et al. [29], the fourth harmonic error of mass *M*_4_ can be decreased by chemical etching at four antinodes along the lower frequency axis of *n* = 2 resonant mode, as is shown in Figure 2a. The spherical coordinate (*r*, *θ*, *φ*) is used to describe the resonator. In Figure 2b, *h* stands for the immersion depth of the resonator into the etching solution, *δ* (0° < *δ* < 90°) is the inclination angle and *α* stands for the zenith angle between the radius-vector from origin *O* to the opposite end points *E*_1_ and *E*_2_ of the edge being etched.

The mathematical relationship among *δ*, *α*, *φ* is:

(13) $$\cos\alpha =1-\frac{h}{R}\sin\delta ,\;$$

(14) $$\theta (\varphi ,\alpha ,\delta )=\left\{ \begin{array}{l} \arcsin(\frac{\tan^{2}\delta \cos\alpha \cos\varphi +\sqrt{1+\tan^{2}\delta (\cos^{2}\varphi -\cos^{2}\alpha )}}{1+\tan^{2}\delta \cos^{2}\alpha }),\left|\varphi \right|\leq \alpha \\ \pi /2,\left|\varphi \right|>\alpha \end{array}\right..\;$$

There is a mathematical constraint between the etching depth *h* and the inclination angle *δ*:

(15) $$0<h\leq \left\{ \begin{array}{l} 2R\sin\delta ,\delta \leq \pi /4,\\ R(\sin\delta +\cos\delta ),\delta >\pi /4. \end{array}\right.\;$$

The total mass to be removed takes the form:

(16) $$m_{4}(\varphi ,\alpha )=4\rho dR^{2}[C_{0}(\alpha )+C_{4}(\alpha )\cos4\varphi ],\;$$

where *ρ* is the density of the resonator, *d* is the thickness of the layer to be removed and *C*_0_, *C*_4_ are determined by the formulae:

(17) $$\begin{array}{l} C_{0}(\alpha )=\frac{1}{2\pi }\int_{0}^{2\pi }\cos\theta (\varphi ,\alpha )d\varphi ,\\ C_{4}(\alpha )=\frac{1}{\pi }\int_{0}^{2\pi }\cos\theta (\varphi ,\alpha )\cos4\varphi d\varphi . \end{array}$$

Fox’s research [9] provides guidance to decide the direction to remove or add mass, and Equation (11) is derived to predict the amount of mass approximately. Here, we only show the way to remove mass, and Fox has proved that to add mass has similar effects. The method proposed by Basarab et al. [29] is cited to design the chemical etching procedure. Subsequent simulation and experiments will further verify the correctness of those conclusions.

## 3. Simulation Experiment

The outer surface of a perfect hemispherical resonator is excavated with a groove, then an imperfect hemispherical resonator model (H00) is created and eigenfrequencies of the *n* = 2 mode are calculated by using the COMSOL Multiphysics software. Table 1 shows the material properties of the imperfect resonator model.

**Figure 3:**
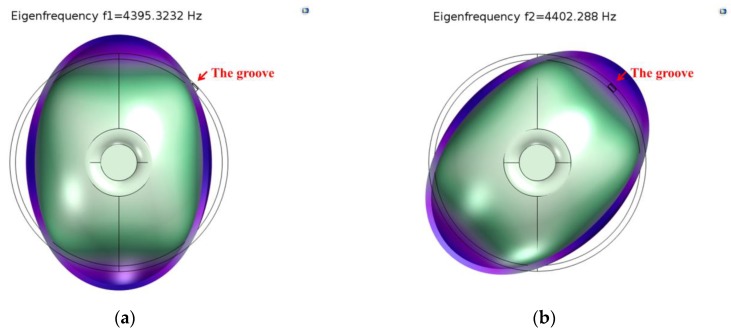
Eigen frequencies of the *n* = 2 mode before etching. (**a**) *f*_1_ = 4395.3232 Hz; (**b**) *f*_2_ = 4402.288 Hz.

We define a fixed constraint on the external surface of the supporting substrate, and analyze the eigen frequency of the resonator under structural mechanics modulus. According to the simulation results, eigen frequencies of the *n* = 2 mode were 4395.3232 Hz and 4402.288 Hz. The modal contour is shown in Figure 3, and the initial frequency split is 6.9648 Hz.

**Figure 4:**
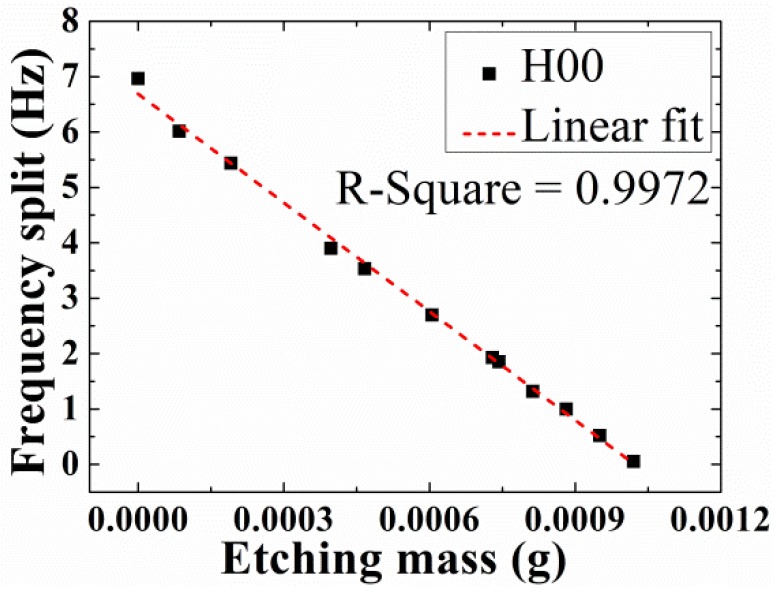
The frequency split of H00 change with etching mass.

The targeted frequency split is around 0.05 Hz. The masses are removed from the four antinodes of the lower-frequency axes on the edge of the resonator little by little, as Figure 1b shows. The frequency split changes linearly with the removal mass, as shown in Figure 4. The absolute value of the slope on the figure is about 6.543 Hz/mg. As the initial total mass is 4.460881g and simulation results indicate that *α*_2_ equals to 0.355, with a linear-fit R-square [30] of 0.9972, according to Equation (11), the theoretical slope *K* is nearly 5.204 Hz/mg.

**Figure 5:**
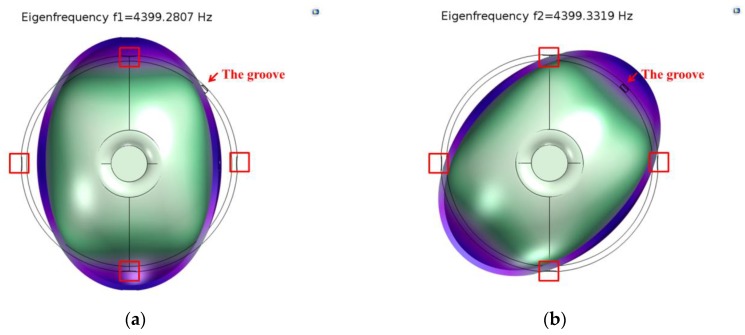
Eigen frequencies of the *n* = 2 mode after etching. (**a**) *f*_1_ = 4399.2807 Hz; (**b**) *f*_2_ = 4399.3319 Hz.

In the last step of trimming, when the removal mass is 0.00102 g, two eigenfrequencies are 4399.2807 Hz and 4399.3319 Hz, and the frequency split is decreased to 0.0512 Hz, as shown in Figure 5, where the four red squares denote the four antinodes to remove the mass. 

Simulation results show that removing masses from the antinodes of the lower-frequency axis can decrease the frequency split. Equation (11) can be used to predict the removal mass approximately, and it reveals that removal mass is linearly related with the frequency split, which is consistent with results obtained by simulation. This rule can be used in experiments, and small amount of masses can be removed step by step to obtain reliable data to calibrate the slope, thus achieving the goal of decreasing the frequency split to a certain value.

## 4. Frequency Tuning Procedure and Results

**Figure 6:**
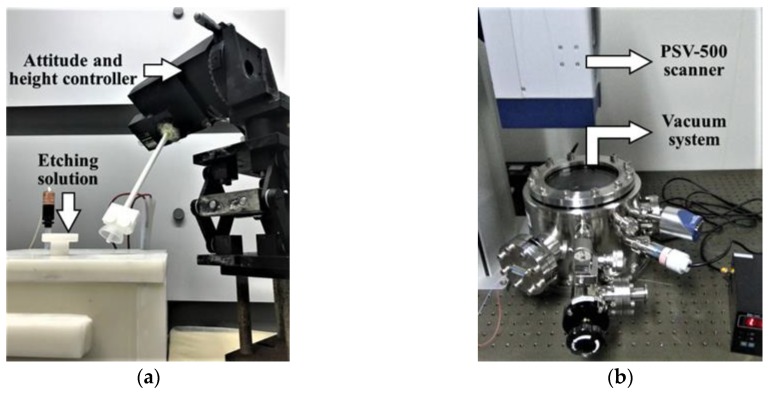
Experimental setup. (**a**) Etching system; (**b**) Measurement System.

An experimental setup was designed as shown in Figure 6. Figure 6a shows the etching system, including an attitude and height controller and the etching solution, which is composed by 10:1 in volume ratio 40 wt % NH_4_F and 49 wt % HF, and Figure 6b shows the measurement system, including a scanning vibrometer (PSV-500, Polytec, Irvine, CA, USA) and a vacuum system, which has been demonstrated in detail in our previous studies [31,32].

**Figure 7:**
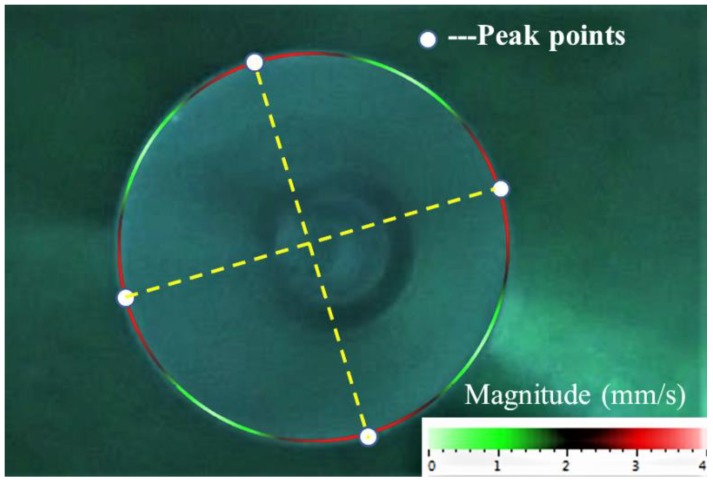
Vibration pattern of the *n* = 2 resonant mode. The four white dots denote the measurement points with the largest vibration velocities, which in the end indicate the etching position to start with. The annotation of the lower right corner shows different magnitudes of vibration velocity.

First, the frequency split and quality factor of the resonator were measured by the PSV-500 and the starting position of the chemical etching was determined. The measurement steps were as follows. In the beginning, scanning points were set around the rim of the resonator and the PSV-500 was used to determine the resonant frequency through a frequency-sweeping process and the frequency and quality factor were simultaneously obtained through the fitting software. Then the vibration velocity of each point was recorded while the resonator was at resonance. Here, four peak points were the points of concern, as they would indicate the etching position to start with in the end. As shown in Figure 7, the annotation of the lower right corner shows that different colors correspond to different vibration velocities. Next we turned the resonator through a rotating platform to find the maximum velocity of the vibration antinode, which indicated that the exciting orientation was along one of the two principle axes. Following the same steps, the other axis could be easily found. In this way, we located the four antinodes of the lower-frequency mode and marked them for later immersion. The initial mass of resonator was 3.4899 g measured by a FA1004 balance (LICHEN, Shanghai, China) with a repeatability of 0.0001 g.

Then the chemical balancing procedure was carried out in the following steps. Four marked antinodes were immersed into the chemical etching solution continually. Here we set *δ* = 53°, *h* = 0.5 mm, with a Φ-30 mm resonator, *C*_0_ and *C*_4_ were 0.0016 and 0.0029, respectively, according to Equation (17). The inclined angle we chose was not the angle that Basarab et al. [29] suggested, because the rate of removing masses was supposed to be slowed down. At each immersion, the resonator was rotated by 90° around its symmetrical axis, which was realized by an electric rotary table. The etching depth adjustment was achieved by a lifting platform, and the etching area was controllable. The etching solution was kept at about 80 degrees centigrade to ensure the proper etching rate. After etching, deionized water and ethanol were used to clean up the resonator. In the end, the frequency split (∆*f*) and quality factor (Q) of the resonator was again measured by the PSV-500. The way to clean up the surface of the resonator and to keep the temperature unchanged is quite challenging, and it’s not easy to control the removal mass that precisely.

**Figure 8:**
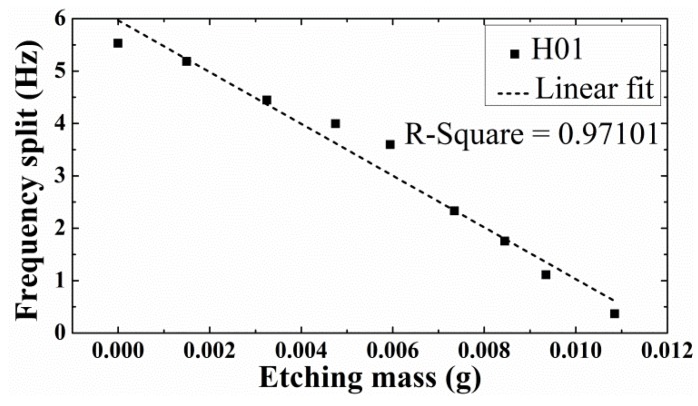
The frequency split change of H01 with its etching mass.

Among several hemispherical resonator samples, resonator 01 (H01) with the largest initial frequency split (5.529 Hz) was chosen for preliminary trial. Through nine chemical tuning rounds, the frequency split of H01 was reduced to 0.364 Hz, when its mass was removed by 0.01085 g. Figure 8 shows that although errors is still significant at this stage, the frequency split of H01 changes almost linearly with etching mass, with a linear-fit R-square of 0.97101. The frequency split of H01 was effectively reduced by chemical etching.

**Figure 9:**
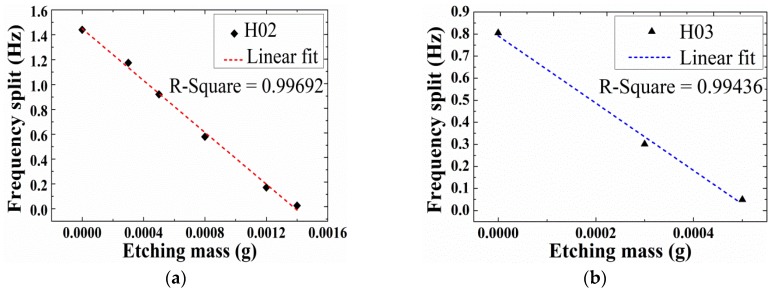
The changes of frequency splits versus etching masses in chemical etching and their linear fits of two hemispherical resonators. (**a**) H02; (**b**) H03.

With favorable results in mind, chemical etching experiments were conducted on other two resonators H02 and H03. The etching solution and etching temperature of three experiments remained the same except for the exact time control of each etching steps in the following experiments. The frequency changing tendency of H02 and H03 is shown in Figure 9. For H02, the removed mass was 0.01085 g in total and ∆*f* was decreased from 1.44 Hz to 0.025 Hz, where the R-square was 0.99692. As for H03, the removed mass was 0.0005 g in total and ∆*f* was reduced from 0.805 Hz to 0.049 Hz, where the R-square was 0.99436.

**Figure 10:**
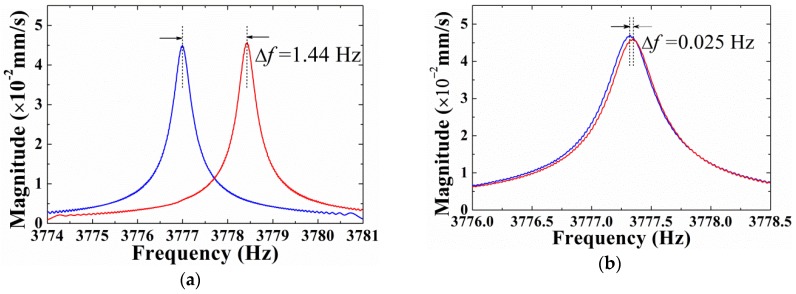
Frequency splits of H02 before and after chemical etching: (**a**) Before chemical etching, ∆*f* = 1.44 Hz; (**b**) After chemical etching, ∆*f* = 0.025 Hz.

Detailed results of each etching round on H02 are demonstrated in Table 2. Resonator H02 had a diameter of 30 mm and thickness of about 0.8 mm. The initial frequency split was 1.44 Hz and the initial mass was 3.4899 g. It was etched for five times, and the immersion durations of which were 30 s, 30 s, 30 s, 30 s and 15 s, respectively. Frequency splits after five etching treatments were 1.175 Hz, 0.921 Hz, 0.58 Hz, 0.171 Hz and 0.025 Hz, respectively. The frequency split variations induced by each etching were 0.265 Hz, 0.254 Hz, 0.341 Hz, 0.409 Hz, and 0.146 Hz, accordingly, and the corresponding mass variations were 0.0003 g, 0.0002 g, 0.0003 g, 0.0004 g and 0.0002 g, respectively. Figure 10 illustrates the frequency splits of the initial and final status of H02, where ∆*f* was reduced from 1.44 Hz, as shown in Figure 10a, to 0.025 Hz, as shown in Figure 10b. Blue and red splines exhibit the frequency responses of the resonator when the resonator was excited at the lower-frequency axis and the higher-frequency axis, respectively. It was shown that, through careful control of experimental parameters, using chemical etching as a method to decrease frequency splits of hemispherical resonators was not only feasible, but also repeatable.

In addition, in contrast to most trimming methods, chemical etching removes masses from the surface of the resonator without changing its stiffness, and altering the quality factor of the resonator significantly. For example, the quality factor of H02 before and after etching were compared in Table 3, where *f*_1_ and *Q*_1_ stand for the frequency and the quality factor of the higher-frequency axis and *f*_2_ and *Q*_2_ stand for the frequency and the quality factor of the lower-frequency axis. Before etching, two quality factors, *Q*_1_ and *Q*_2_, are 10,135 and 10,150 in air, respectively. After etching, they are 10,247 and 10,133, respectively, only slightly different with that before.

Furthermore, theoretical calculations and experimental results were compared in Table 4. The estimated slope refers to the slope *K* in Equation (11). For three resonators, α_2_ almost equals to 0.034 according to test results, and the discrepancy between simulation and experiment is considerable. Resonator H01 has the poorest linearity between frequency splits and etched masses. This might be ascribed to the lack of proficiency in the procedure and lack of control on the processing time, etc. For H02 and H03, the linearity between frequency splits and etched masses improved significantly, however, they still have differences between estimated slope and actual slope of 0.04 Hz/mg and 0.628 Hz/mg, respectively. Although the frequency split changes linearly with the etched mass, the relationship between the frequency split and the mass to be removed is highly resonator-dependent.

Many factors may influence the etching effect, such as temperature stability, small changes of chemical etching solution concentration, the diffusion effect of the etching solution on the surface of the resonator, position accuracy of the four antinodes, etc. Efforts were made to improve the preciseness of chemical etching focus on the control of the etching height, the etching temperature, the concentration of the solution, and the inclined angle, even though it is not compatible to ion beam etching. Moreover, lowering the temperature of the etching solution, thus slowing down the chemical reaction, can help control the precision of the process and we will try it in future work. In general, through determining the frequency split, the principle axes and the removal mass by the approximate formula, then removing certain masses by chemical etching, the frequency splits of hemispherical resonators can be eliminated. The strength of chemical etching method lies in its high efficiency and simplicity, and it can be used as a pretreatment method.

## 5. Conclusions

We have demonstrated with experiments the decreasing frequency splits of fused silica hemispherical resonators by the method of chemical etching for the first time. Experimental results are in accordance with the research of Basarab et al. [29]. We conclude that chemical etching can decrease frequency splits of hemispherical resonators effectively, without damaging their quality factors. By means of chemical etching, the frequency split can be decreased to below 0.05 Hz. Moreover, chemical etching is convenient to implement, requires less time and labor input, and costs much less compared with mechanical treatment, laser ablation, ion-beam etching, etc. From all the above, the chemical etching method can be regarded as an effective trimming method for medium accuracy hemispherical resonator gyroscopes. However, the optimization of the chemical procedure, including the etching parameters, chemical solution and environment control, merits further research.

## Figures and Tables

**Table 1:** The material properties of the imperfect hemispherical resonator model.

Properties	Young’s Modulus (*E*)	Density (*ρ*)	Possion’s Ratio (ν)
**Amount [Unit]**	71.7 [Gpa]	2203 [kg/m^3^]	0.17

**Table 2:** The frequency split and mass change of H02 of each chemical etching.

Etching Rounds	Etching Time ∆*t* (s)	Frequency Split ∆*f* (Hz)	∆*f* Variation ∆(∆*f*) (Hz)	Mass Variation ∆*m* (g)
1	0	1.44	0	0
2	30	1.175	0.265	0.0003
3	30	0.921	0.254	0.0002
4	30	0.58	0.341	0.0003
5	30	0.171	0.409	0.0004
6	15	0.025	0.146	0.0002

**Table 3:** Quality factors of H02 before and after chemical etching along the higher-frequency axis and the lower-frequency axis.

Status	*f*_1_ (Hz)	*Q*_1_	*f*_2_ (Hz)	*Q*_2_
Before etching	3778.430	10,135	3776.690	10,150
After etching	3777.344	10,247	3777.319	10,133

**Table 4:** The contrast between the estimated slope and actual slope in experiments of three resonators.

Resonator No.	Initial Mass *m*_0_ (g)	Initial Frequency *f*_0_ (Hz)	Frequency Split *∆f* (Hz)	Final Frequency Split *∆f’* (Hz)	Estimated Slope (Hz/mg)	Actual Slope (Hz/mg)
H01	3.7324	4104	5.529	0.364	1.099	0.493
H02	3.4899	3778	1.440	0.025	1.082	1.042
H03	3.5542	3190	0.818	0.049	0.897	1.525
